# On Non-Mechanical Restraint in the Treatment of the Insane

**Published:** 1854-10-01

**Authors:** 


### Art. VI.—ON NON-MECHANICAL RESTRAINT IN THE TREATMENT OF THE INSANE.

In the Appendix (G) to the Eighth Annual Report of the Commissioners in Lunacy, just published, we find recorded the answers to a circular issued last year by the Commissioners, and addressed to the superintendents and medical proprietors of the principal lunatic asylums, registered hospitals, and licensed houses in England and Wales, requesting information as to the employment or disuse of instrumental restraint and seclusion in the treatment of the insane.

Before proceeding to analyze this important body of conflicting evidence upon a much-vexed question, we would make a few preliminary observations. In the first place, we think we may take, with justice, exception to the term used in the Report, when referring to this subject,—*viz.*, that of "*instrumental* restraint." This phrase conveys to the uninitiated and popular mind a very erroneous idea of the nature and character of the kind of restraint many humane and conscientious medical men engaged in the management of asylums and the treatment of the insane, consider themselves justified in using in certain urgent and peculiar cases of insanity. The word "mechanical" restraint is well understood, but the term "instrumental" restraint immediately suggests to the mind iron-chains, leg-locks, bolts, and other barbarous modes of confining the limbs of the insane, adopted during the dark ages. God forbid that those days ever should return! With a view of estimating correctly the degree of value that should be attached to the evidence we propose analyzing, it will be necessary to bear in mind the following points:—In the first place, many of the gentlemen who have forwarded replies to the Commissioners, and who have expressed an unqualified opinion in favour of non-restraint, are men of but limited experience, having but for a short period been practically engaged in the treatment of the insane. Again, a few of the medical men who answered the circular issued by the Commissioners, are in the habit of admitting into their houses a limited number (to use a phrase familiar to most of those who read the advertisement columns of *The Times*) of "nervous invalids," a quiet class of patients not at all likely to require the application of mechanical restraint in their treatment. The evidence of these gentlemen is not, therefore, of much value, *quoad* the question at issue. Secondly, we are bound to consider what we conceive to be an important element in relation to the matter under review: viz. that many who have recorded their opinion in favour of unconditional non-restraint would, from their position, hesitate in giving utterance to views adverse to those that have so tenaciously fastened themselves upon the public mind.

A gentleman anxious to obtain *status* in this department of practice, and not having age or experience to guide him to a scientific and right deduction, would pause before committing himself to an opinion opposed to popular prejudices. He would naturally hesitate in adopting what the public have sedulously been taught to consider as the *in*-humane side of the question; and rather than run counter to this feeling, would at once join the ranks of the extreme party, and throw up his cap in favour of non-restraint. The recorded opinions of this class should consequently be taken with considerable limitations. Thirdly, it is necessary to recollect that many whose answers are published by the Commissioners have, from an early period of their career, pledged themselves to *ultra* opinions upon this question. It is not at all probable that any of these gentlemen would easily be induced to abandon a dogma upon which their whole reputation is based. Fourthly, it will be impossible to draw any sound conclusions from the evidence before us, without being fully acquainted with the *substitutes that have been used for the strait-waistcoat and other modes of mechanically restraining the insane.* Have not the frequent administration of nauseating doses of the tartrate of antimony, the shower and cold bath, in several asylums taken the place of mechanical restraint, producing, as can be readily conceived by those conversant with the pathology of insanity, the most disastrous consequences? The use of the milder forms of mechanical restraint in cases of acute and dangerous insanity can do little or no permanent injury, but *the repeated and continuous exhibition of tartar-emetic, chloroform, and stupefying doses of opium, with the view of subduing the muscular violence of the insane, and thus reducing them to a manageable condition, and obviating the necessity for mechanical restraint, may do serious and irremediable mischief;* and for this obvious reason, that the patient is compelled to take medicines which greatly depress the nervous system at a time when everything should be done to sustain the *vis vitæ*, and give increased impetus to the nerve force. It does not require much sagacity to reduce, by these means, a violent lunatic to a state of comparative composure and quietude; but we would caution all engaged in the anxious and responsible duties of treating the insane, against the adoption of a course alike dangerous to life, and perilous to reason. All who have recorded their opinion in favour of unconditional non-restraint, and who declare that no case of insanity can possibly arise in which it will be necessary, should be compelled to state to what extent they use the shower, cold-bath, opium, and tartar-emetic, &c., before we can attach any scientific importance to their view of the matter in dispute. Having made these cursory remarks, we at once proceed to our proposed analysis. After carefully examining all the returns made to the Commissioners, we have classified the men agreeably to the following form:—

1. Advocates for a qualified use of mechanical restraint.
2. Advocates for the total abolition of restraint.
3. Those who do not use restraint, but who give no opinion on the abstract question.
4. Advocates for restraint in surgical cases.
5. Those who give a qualified opinion on the subject of restraint.

The subjoined tabulated statement will be found accurate in its details:

*Advocates for a Qualified Use of Mechanical Restraint.*

J. Harris.
B. F. Mathews, Bedford Co. Asylum.
John Millar, Bucks Co. Asylum.
John Bucknill, M.D., Devon Co. Asylum.
Richard Oliver, M.D., Salop and Montgomery Asylum.
John Wilks, Stafford Co. Asylum.
John Thurnam, M.D., Wilts Co.
Samuel Hill, York, North and East Riding. 144.
Thomas Green, Birmingham Borough Asylum.
Abs. Stansbury, Bristol Asylum.
F. W. Casson, Hull Borough Asylum.
B. Formby, M.D., Liverpool Asylum.
C. M. Gibson, Bethel Hospital, Norwich.
W. Allen, Warneford Hospital.
John Kitching, The Friends' Retreat, York.
James Phillips, Bethnal House.
D. M. Maclure, Earl's-Court House, Brompton.
D. T. Roy, Montague House, Hammersmith.
J. H. Paul, Camberwell House.
A. J. Sutherland, M.D., Otto and Blackland House.
John Bush, Clapham Retreat.
Ed. W. Monro, M.D., Brook House, Upper Clapton.
F. Winslow, M.D., Hammersmith.

Bowling and Halford, Normand House, Fulham.
F. Oxley, M.D., London House, Hackney.
John C. S. Nicoll, Elm Grove, Hanwell.
J. W. Holgate, Hindon House, Middlesex.
Henry Armstrong, M.D., Peckham House.
J. R. Atkins, M.D., Stoke Newington.
W. T. Spencer, Stoke Newington.
L. Glenton, Benshaw Asylum.
R. Davis, Wrickenboy Asylum.
B. Barkus, M.D., Gateshead Asylum.
H. Griesback, M.D., Dunston Lodge.
T. Tomkin, Witham, Essex.
James Cornwall, Farnford Retreat.
C. M. Burnett, M.D., Westbrook, Alton.
S. Millard, Whitchurch House, near Monmouth.
F. A. Young, North Grove House, Hawkhurst.
R. F. Ainsworth, Blakeley House, Manchester.
W. H. Parsey, Warwick Co. Asylum.
E. Simpson, York Hospital.
J. Smith, Hadham Palace Asylum.
D. Noble, M.D., Clifton Hall.
J. B. Whitehead, Haydock Lodge.
F. Willis, Shillingthorpe House.
H. Landor, Heigham Retreat, Norwich.
W. P. Nicholls, F.R.C.S., Heigham Hall, Norwich.
W. H. Ranking, M.D., Heigham Hall, Norwich.
J. F. Watson, Heigham Hall, Norwich.
D. Mackintosh, M.D., Newcastle-upon-Tyne Asylum.
R. Mallam, Hooknorton.
J. H. Norton, Amroth Castle, Tenby.
F. and C. Fox, Brislington House, Bristol.
J. Terry, Bailbrook House, Bath Easton.
W. E. Gillet, Fairwater House, Taunton.
J. F. Woody, The Moat House, Tamworth.
G. F. Furnival, Great Foster House, Egham.
Charles Summers, Great Foster House, Egham.
J. R. Stedman, Lea Pale House, Guildford.
C. H. Newington, M.D., Ticehurst.
S. Newington, M.D., Ticehurst.
G. Bodington, Driffold Asylum.
J. Warwick, Laverstock House, Salisbury.
J. Nash, M.D., Kingsdown House, Box.
J. Anningson, Marfleeet Lane Retreat, Kingston-upon-Hull.
G. P. Smith, M.D., Castleton Lodge.
T. Allis, Fern Hall, Osbaldwick.
J. W. Metcalfe, Acomb House, York.
S. Nelson, Grove House, Acomb.
J. Atkinson, Heyworth Asylum, York.

*Advocates for the Total Abolition of Mechanical Restraint.*

R. Lloyd Williams, M.D., Denbigh Asylum.
George T. Jones, M.D., Denbigh Asylum.
John Hitchman, M.D., Derby Co. Asylum.
Donald Campbell, M.D., Essex Co. Asylum.
John D. Cherton, Lancashire Asylum, near Rainhill.
James Holland, Lancashire Asylum, Prestwich.
John Buck, Leicester and Rutland Asylum.
J. T. Allen, Monmouth Asylum.
R. Foote, M.D., Norfolk Co. Asylum.
F. D. Walsh, Lincoln Co. Asylum.
Henry Stevens, St. Luke's Asylum.
W. C. Hood, M.D., Bethlehem Hospital.
Edwin Wing, York House, Battersea.
Alonzo H. Stocker, Grove Hall Asylum, Bow.
W. F. H. Ramsay, M.D., Wyke House, Brentford.
W. D. Williams, Pembroke House, Hackney.
E. L. Bryan, Hoxton House.
E. V. Hensey, M.D., High Beech Asylum.
Charles Broughton, Vernon House, Britton Ferry.
James George Davey, M.D., Northwood, Bristol.
R. G. Hill, Eastgate House, Lincoln.
W. H. Hugo, Longwood House, Bristol.
G. Scrase, Ringmer House, Lewes.
W. Berrow, Duddeston Hall, Birmingham.

J. Kirkman, M.D., Suffolk Co. Asylum.
W. Dickson, Manchester Lunatic Asylum.
W. C. Finch, M.D., Fisherton House.
Charles Snape, Surrey Co. Asylum.
R. W. Diamond, M.D., Surrey Co. Asylum.

*Medical Superintendents of Asylums who do not use Restraint, but who give no Opinion on the Abstract Question.*

D. F. Tyerman, Colney Hatch Asylum, Middlesex.
W. C. Begley, Hanwell Male Division, Middlesex.
J. Stocker, Guy's Hospital Lunatic Ward.
J. B. Steward, M.D., Southall Park.
W. Wood, M.D., Kensington.
A. G. Kerr, Regent's-park.
J. O. Rumball, St. Alban's.
Smith Slatebrook, Tue Brook Villa, Liverpool.
W. Cooper, Norwich Infirmary Lunatic Asylum.
Thomas Pritchard, Abington Abbey, Northampton.
J. Pownall, M.D., Calne, Wilts.
Thomas Laycock, M.D., Gate, Helmsley.
Caleb Williams, Terrace House, near York.

*Advocates for Restraint in Surgical Cases.*

W. Ley, Oxfordshire and Berks Asylum, Littlemore.
Robert Boyd, Somerset Co. Asylum.
Harrington Tuke, M.D., Manor House, Chiswick.
J. Conolly, M.D., Hanwell.

*Medical Superintendents who give a Qualified Opinion on the subject of Non-restraint.*

R. Langworthy, Plympton Asylum.
W. H. Pursey, Warwick Co. Asylum.

In the first place, we proceed to give the opinions of those who advocate the *total* abolition of all mechanical restraint. Dr. Lloyd Williams and Mr. G. T. Jones, of the Denbigh Lunatic Asylum, say that, "Since the opening of the asylum in 1848, we have never had cause to deviate from the uniform and consistent practice of avoiding the slightest mechanical restraint in the treatment of the insane, beyond the occasional use of the padded room in cases of extreme violence; and the seclusion has been confined to as few cases as possible, and for as short periods as can be avoided. We have sedulously endeavoured to impress upon our attendants that they are never to exhibit the slightest exhibition of temper, or resentment, for conduct however violent or provoking, and that they are to practise 'the law of kindness,' as the code by which the confidence of their patients is to be gained, and their violence subdued."

When speaking of the substitutes for mechanical restraint, they observe that, "In some cases of excessive maniacal violence, we have successfully resorted to immersion in the cold bath, and in other cases to the application of a continuous stream of cold water upon the head, whilst the patient is sitting in a warm bath. The shower-bath is also found of much use in producing tranquillity in similar cases."

"Immersion in the cold bath," and the "application of a continuous stream of cold water upon the head," are questionable modes of procedure, if adopted *merely* to "produce *tranquillity*," and subdue "excessive manical violence;" if used as a curative means in properly selected cases, well and good.

Dr. Hitchman, of the Derby Asylum, says that since 1843, "I have not sanctioned the use of any kind of mechanical appliance to control the limbs of any refractory or suicidal patients, and I have not met with any case in which, with good attendants, and a well-arranged building, restraint appeared necessary; on the contrary, patients have been brought to the various institutious which have been under my care, who had been rendered more violent and more suicidal by the means taken to control them prior to admission."

Dr. Campbell, of the Essex Lunatic Asylum, uses no mechanical restraint, he observes:—"I feel justified in stating it as my opinion, that personal restraint is in no case necessary for the treatment of insanity in a properly constructed asylum, and that in all cases it is prejudicial."

This gentleman is in favour of seclusion. He says, with a view of "bringing the health of the patient into the best possible state, constant occupation and amusement afford the most powerful means of curing and alleviating the disease."

Mr. Cleaton, of the Lancashire Asylum, Rainhill, has not found mechanical restraint necessary, since the opening of the institution in 1851. He remarks that, "As far as the experience of this institution goes, the best substitute for seclusion, generally speaking, appears to be out-door occupation; and it has been a common practice here with regard to artisan patients, when a paroxysm of excitement comes on in the course of chronic mania, rendering them unable to follow their special avocation, to send them into the land to be employed in simple agricultural occupation, such as wheeling soil, making or repairing roads, &c., and when the attack passes away, to allow them to return to their workshops."

Mr. Holland, of the Lancashire Asylum, Prestwich, says:—"Mechanical restraint has been applied in this asylum only once since it was opened, upwards of three years since, and this would not have happened had that part of the establishment principally used for the treatment of maniacal patients been ready for occupation when the institution first admitted patients. Seclusion is rarely resorted to, except in instances of acute or epileptic mania, of which we have a great number, and in such cases I consider seclusion to be a very essential part of the treatment."

Mr. John Buck, of the Leicestershire and Rutland County Asylum, has abolished all mechanical restraint. We may say the same of Mr. Tyerman, of the Colney Hatch Asylum.

Mr. J. S. Allen, of the Monmouthshire Asylum, says, that since December 1851, "362 patients have been admitted. The great majority of the cases had been for many years insane, and a large proportion (viz. 51 cases) were complicated with epilepsy. Mechanical restraint or coercion has not been used in any case, and the want of it has not been felt. The general effects of non-restraint on the patients themselves, as well as on the attendants, have been salutary. The patients, with few exceptions, however deficient in intellect they may be, know that restraint cannot be used towards them, and this alone has a tranquillizing effect, as no class of sane persons are more morbidly sensitive as to receiving harsh or unfair treatment than the insane."

Dr. Foote says:—" I have never seen mechanical restraint produce any beneficial effect in the treatment of mental diseases, but have seen many cases greatly relieved by the removal of restraint."

Mr. Charles Snape, of the Surrey Asylum, says:—" Mechanical restraint is never employed where the arrangements are good, and in a properly-constructed asylum I believe this system to be quite superfluous."

Dr. Diamond, of the same asylum, says:—" I believe that any person who would now use personal restraint or coercion *is unfit to have the superintendence of an asylum!*"

This our readers will consider to be a very bold opinion.

" I have," he continues, " at the present time upwards of 520 female patients under my immediate charge; and, during the past five years, have admitted more than 800 cases. In not a single instance has any restraint been used."

Dr. Kirkman, of the Suffolk County Asylum, says:—" The Suffolk County Asylum has been, for the last *twenty-three years*, under the same resident Medical Superintendent, and throughout the whole of that period, the mildest system of treatment has been ceaselessly carried out. All instruments of mechanical restraint were destroyed more than twenty years ago, and they have neither been used or required ever since. It is our uniform practice to enter every case of temporary separation, if a patient is placed only a few minutes in a room, and this is not very often needed, and consequently rarely done. Seclusion in a padded room we have found very seldom necessary. I may state as a general fact, built upon lengthened experience, that association with the insane, and constant supervision over them, will secure that moral control which supersedes the necessity of any measures, which, though they may be used only as preventives against injury to the patient themselves, have a semblance of restriction about them. The mildest treatment is unexceptionably the most successful."

How is it that Dr. Kirkman, having abolished mechanical restraint for a period *of twenty years*, should not be placed in the foremost ranks, or even at the *head* of those who, like Mr. Hill, have claimed the credit of having originated the "non-restraint system" of treating the insane in this country? Surely Dr. Kirkman is entitled to a testimonial, and after his death (which we hope is far distant) should have a statue erected to his memory!

Mr. Dixon, of the Manchester Lunatic Asylum, says:—"With regard to mechanical restraint, I substitute for it exercise, under carefully-selected attendants, in the grounds and fields belonging to the institution."

Mr. Walsh, of the Lincoln Asylum, says:—"There has been no mechanical restraint used in this asylum since the 17th of April, 1840. I have seen manual restraint, or the holding of maniacal patients, practised here, and believe it to be a most cruel kind of restraint. It is impossible to hold a strong and refractory patient for a long time without injury and danger both to the patient and attendants. During the time that this asylum has been managed without mechanical restraint, seclusion, or manual restraint, I have not seen any cases in which I consider such restraints would have been beneficial, but probably injurious."

Mr. Stevens, of St. Luke's, says:—"I believe the entire abolition of every kind of mechanical restraint to be the most humane, the most efficacious, and, speaking generally, the safest plan of treatment; on the whole, less liable to objection than any other, and perfectly practicable in a well-regulated and properly-conducted institution."

Dr. W. C. Hood, resident-physician at Bethlehem, says:—"No form of mechanical restraint whatever is resorted to in this hospital. The 'Non-restraint system,' as it is called, is adhered to, because it is found to be attended with the best and happiest results; whereas the confinement by straps, belts, or gloves rather increases the excitement, irritates the patient, reduces the necessity of vigilant personal attendance, and not infrequently induces chronic or permanent mania. If, during great excitement (which is generally paroxysmal), the patient cannot be soothed by kindness, temporary seclusion in the bedroom, or, if dangerous, in the padded room, will usually be found sufficient; if not, the administration of sedatives. I prefer giving a full dose, and repeating it in four or six hours. Should the excitement be persistent, and the patient of robust habit, the sedative effect of the anodyne will be of little use. In such cases I have found much value in prescribing small and repeated doses of antimony, a third or half a grain three times a day in solution. I am of opinion that the excitement is consequent upon irritation, and not inflammation, and therefore, unless strongly indicated, always eschew depletion."

Mr. Wing, of Battersea, says:—"My opinion is, that with a sufficient number of proper attendants, the use of such means may be entirely dispensed with."

Mr. Stocker, of Grove Hall, Bow, says:—"I am convinced that the use of mechanical restraint under any circumstances is both unnecessary and unjustifiable."

Dr. Ramsay, of Wyke House, observes: —"I have never myself used or advised mechanical bodily restraint, and I am convinced, that where it is employed all moral treatment is neutralized, and that it militates against the acquisition of the patient's confidence, which last ought to be the first endeavour of the physician who undertakes the treatment of the insane."

Dr. W. D. Williams, says:—"By contrivance, management, and watchfulness, therefore, aided by a staff of kind, intelligent, and quiet, yet active and energetic attendants, it is my opinion that seclusion and restraint may be ultimately rendered unnecessary; and I consider that by the same means bad habits may generally be cured."

Mr. Bryant, of Hoxton House, says:—"That during the last two years and a-half every article of restraint has been removed from the house."

Mr. Kerr, of Regent's Park, says:—"No kind of mechanical restraint is ever resorted to. I have always found that kindness, blended with firmness, is the most efficient mode of allaying excitement, however violent."

Dr. Hensey, of High Beach, says, That mechanical restraint, or over-rigid seclusion, are the sure means of making maniacs of severe cases, and of greatly aggravating the mildest form of insanity, by fretting and irritating minds already sufficiently excited. My plan of treatment is to allow all to do pretty much as they like, and to go where they like, always under the eye of a sufficient number of attendants, who are ready to interfere only when any very great irregularity is attempted, and even then it is generally sufficient to merely catch their eye."

Mr. Broughton, of Britton Ferry, speaks boldly out upon the question. He says:—"In the treatment of the insane, it is difficult to imagine a case in which mechanical coercion can be deemed advisable, or even allowable. No instances have fallen under my observation which could seem to justify its employment, or hold out the attainment of any desirable end."

Dr. Davey, of North Woods, Bristol, says:—"There must be no mechanical restraint, nor perpetual seclusion in an isolated apartment, to take the place of that kind and discriminate care and attention so indispensable to the relief or care of him mentally afflicted." He further adds:—"*There are few, indeed, among the insane, who are wholly lost to the higher and purer feelings of our nature, whose affections are, one and all, blighted and perverted, or whose emotions are quite gone astray, and altogether beyond restoration and repair; the*

*fact will supply the superintendent with the requisite data as to the treatment.*"

Having made the preceding just remarks, how could he commit himself by making the following observation:—"One remark I would venture to make, viz., *that there must be, as a rule, no positive indulgence shown towards the insane.* Whatever may be the nature of the caprice or irregularity which pervades the mind of a patient, in what way soever his perverted feelings and desires may be manifested, however much or little his emotions may be disordered, it is the duty of the physician to strive to amend the indications of disease in him, and not to humour the various backslidings of his mental nature."

If, as he asserts, there are "few indeed among the insane who are lost to the higher and purer feelings of our nature," &c., surely Dr. Davey must meet with many cases in his practice, in the treatment of which it would not only be *necessary*, but *humane*, to bring them within the range of "positive indulgences."

Mr. R. G. Hill, of Lincoln, says:—Perfect "non-restraint is practicable, for it has been well tested; it is humane, as all must acknowledge; it contributes to the comfort, the cheerfulness, and the recovery of the insane. It is also safe, for no serious or fatal accident has occurred in consequence of it. Constant surveillance has prevented this; it soothes the patient, keeps his angry and revengeful passions at rest, gives him the power to assist himself, and thereby prevents his falling into habits of hopeless filth and misery; and I venture to pronounce of it, that it is the system which must and will ultimately prevail in every asylum."

Mr. Hugo, of Long Ashton, Bristol, has had no occasion to use restraint or seclusion during the few weeks he has had the management of that asylum.

Mr. Scrase, of Lewes, says:—"I am quite convinced that the non-restraint system is the preferable treatment; and I do not intend to have recourse to restraint again, believing that it may be entirely dispensed with."

Mr. Berrow, of Duddeston Hall, near Birmingham. This gentleman says:—"I have, during the last four years, been enabled to bear testimony to the great advantages arising from the abolition of mechanical restraint, with a full assurance that such measures greatly improve the condition, health, and comfort of the insane."

Dr. Finch, of Fisherton House Asylum, says:—"The system of treatment pursued in this asylum for the insane is, upon the admission of a patient, to remove all mechanical restraint from his person, and at once to let him have as much personal freedom, in-doors and out, within the precincts of the establishment, as the nature of the case will permit, with a due regard to his own safety and that of others."

Dr. Laycock, of York, in an interesting report, advocates non-restraint. He says that, "The non-restraint system of treating the maniacally-violent should be founded wholly in the psychology of the instincts and emotions. As to the use of persons or of mechanical appliances, when physical force is absolutely necessary (and such cases must inevitably occur), my experience is in favour of the latter. Personal restraint in the sane excites resistance, and the desire to attack the restrainer; how much more in the insane, in whom the disposition to attack and resist is morbidly developed already! The manner and expression of the attendant during the struggle with the maniac must also act as a powerful stimulus; for philosophy and experience convince us that he cannot remain perfectly free from emotion, when his corporeal energies are called forth to resist the struggles of a violent maniac, however well disciplined, so potent and ever active are the stimuli to these instinctive emotions and passions."

We now proceed to give a *resumé* of the evidence in favour of a qualified application of mechanical *restraint* in the treatment in peculiar and special cases of insanity.

Messrs. J. Harris and B. F. Mathews, of the Bedford Asylum, observe:—"We believe the objection to restraint is not well founded, but arises from the abuse, and not the proper use of it; and that the evils incident to the so-called 'Non-restraint' system, are greater than those attached to the treatment we advocate."

Dr. J. C. Bucknill, of the Devon County Asylum, who has returned an able and full report to the circular of the Commissioners, makes the following important admission:—"In the Devon County Asylum, restraint is never employed, except in surgical cases; in these, of course, the same principles must be adopted for the insane as are necessary for the sane, to insure that absolute quietude of parts which is essential for the advantageous conduct of the healing process. It is not denied that cases have occasionally arisen in which it has been difficult in the extreme to avoid the imposition of restraint; for instance, those of suicidal patients who have endeavoured to effect their purpose by thrusting articles of clothing and other substances down the throat, by beating the head against the wall, and by other means, which are scarcely capable of being obviated by any watchfulness on the part of the attendants. A patient is still resident in this asylum, who endeavoured to commit suicide by lacerating the veins of the fore-arm with his teeth, and who bit out from his arm large pieces of flesh in the attempt. Had these efforts continued, it would not have been possible to have avoided the imposition of restraint, except by defending the arm by hard leather sleeves; by restraining the teeth, in fact, instead of the limbs. The occurrence of such cases, however unfrequent they may be, renders it impossible to deny that the imposition of mechanical restraint may, in rare instances, be necessary for the safety of the patient."

Whilst admitting that mechanical restraint *is necessary*, he makes the following startling assertion:—"Mechanical restraint in the treatment of the insane is, like the actual cautery in the treatment of wounds, a barbarous remedy, which has become obsolete from the introduction of more skilful and humane methods, but which may still be called for in exceptional and desperate cases. It may be said that as these cases are so rare, that as large asylums are conducted for many years without one of them being met with, that as they do not appear, they may be considered as if they did not exist."

It occurs to us, that if mechanical restraint be, as Dr. Bucknill describes, a "barbarous remedy," assimilated to "actual cautery" in i's operations, it is not justifiable, under any possible state of circumstances, not even in the surgical and suicidal cases referred to by this able physician as illustrations of the propriety of restraint in certain cases of insanity. We direct particular observation to the following sensible remarks:—"The lunatic is unable, without assistance, to control his actions so that they may tend to his own well-being, and to that of society. He is therefore placed under care and treatment, that he may be restored to the power of self-control; under care, that while this power remains impaired he may be assisted in its exercise. This assistance may come in the shape of a strait-waistcoat, or in the fear of one: or it may come in the sense of duty imposed in the operation of a gentle but effective discipline, of honest pride, desire of approbation or personal regard, or the still nobler sentiments of religion. The first motive, that of fear, belongs to man and the animals, and its exercise is degrading and brutalizing; the latter motives are humane and humanising in their influence, and their development is the true touchstone of progress in the moral treatment of mental disease. It was the brutalizing influence of fear, and the degrading sense of shame, which constituted the true *virus* of mechanical restraints. In repudiating the use of mechanical restraints in the Devon Asylum, the above principle has been kept in view with a jealous anxiety lest the moral effects of restraint should present themselves in some other form. It would seem that it is more easy, or at least more consistent with our nature, to rule by fear than by love. And the annoyances caused by the insane, on their immediate attendants, are hard to be endured without exciting a spirit of retaliation. For this reason the plan of manutension, or holding violent patients for a long time by the hands of attendants, scarcely deserved the name of a reform; and seclusion, injudiciously and harshly employed, is liable to the same objection. If a patient is to be ignominiously thrust into a dark and comfortless cell, and detained there for an indefinite period on the occasion of any outburst of temper or irritability, it may well be doubted whether mechanical restraint does not possess some advantages over such a system; and the French physicians may be perfectly justified in preferring the *gilet* to their own *cellules de force*. But in my opinion, seclusion differs widely from restraint in its capacity for beneficial employment; restraint, except in cases so rare that they may be left out of consideration, is always an unmitigated evil. Seclusion, wisely employed, is frequently an important and valuable remedy. The character of seclusion, as a remedy, has never recovered from the attacks made upon it by the advocates of mechanical restraint. They represented, truly enough, that a patient walking about pleasure grounds, with his arms tied to his sides, was capable of more enjoyment than he would be if shut up in a dark and narrow cell, with all his limbs at liberty. In this objection, the fundamental principle of the new system was overlooked, that neither by restraint, seclusion, nor any other means, was it permissible to inflict upon the insane any unnecessary or avoidable suffering, or any indignity or degrading coercion, whether of a physical or moral kind. But the possible abuse of a thing is no valid argument against its use; otherwise there is no important remedy, medical or moral, which might not be equally objected to."

Dr. Oliver, of the Salop Asylum, says:—"I have never had occasion to employ mechanical restraint in the treatment of the insane, and I have never seen such circumstances as would, in my opinion, justify recourse to such coercion in preference to the practice of seclusion."

After making this declaration, from which one would have imagined that he was an out-and-out advocate for the total abolition of mechanical restraint, he startles us with the following remarks:—"Certain circumstances, as for instance the obstinate refusal of a person to take food, may render it necessary to overcome this reluctance by means of mechanical compulsion, and by the use either of the stomach-pump, or of a flexible tube introduced through the nostril; and it may be advisable to place the individual in such a position at the time of the operation, that he may, as little as possible, be able to make resistance; but beyond this kind of necessity, I can see no good to come from directly restraining the action of the limbs."

We leave Dr. Oliver to reconcile, in a manner most consistent with his view of the question, this obvious discrepancy in his recorded opinion.

Dr. Wilkes, of the Stafford County Asylum, makes the following remarks:—"With every disposition to advocate the disuse of restraint to the utmost extent, I am compelled to admit that the result of my experience in this asylum, up to the present time, leads me to the conclusion, that cases may occur in which its temporary employment may be both necessary and justifiable. Besides the occasional use of some means of confining the hands when feeding patients by means of the stomach-pump, a more prolonged use of restraint was found necessary in two cases which occurred some years since. One of these was a man with so determined a suicidal disposition, that on more than one occasion he nearly effected his purpose by trying to beat his head and face against the walls, to throw himself from tables and chairs, and thrust spoons and other articles down his throat. When first admitted, he was not suspected of having any suicidal tendency, and for some weeks did not show any; as a matter of precaution, he slept in a padded room, and one night he so battered his head with a tin chamber utensil, that he was found nearly dead from loss of blood, and his life was subsequently in much danger from extensive sloughing of the scalp. In this case, it was absolutely necessary to confine the hands to keep any dressings on the head, and after the wounds had healed, and the confinement to the hands had been discontinued, he wore a thickly padded cap for many months. Several years after this, he bit both his little fingers off, and though the suicidal disposition has in a great measure subsided, he is still at times much excited, but does not require any restraint. The second case was one of acute mania in a powerful young man, who refused all food, under the impression that it was poisoned, and imagined that every one who went near him intended to murder him. Every inducement to get him to take food was in vain, and though a sufficient body of attendants under my own inspection attempted to do what was necessary for him, he became so much bruised with holding him in his struggles to assail the attendants, and it was so urgently requisite that food should be introduced into the stomach, that I decided upon confining his hands, and both food and medicine were then readily administered. The result certainly justified the means employed, as the excitement soon subsided, and he recovered rapidly."

In justice to Dr. Wilkes, we should observe—"These were extreme cases, and such as may not occur again for years; and although perhaps 999 cases may be safely dealt with without resorting to any mechanical restraint, still the next which offers may baffle the ingenuity of the greatest advocate of non-restraint, and be one in which the employment of some means of coercion cannot be avoided with due regard to the safety and well-being of the patient."

Mr. W. H. Parsey, of the Warwick County Asylum, says:—"Mechanical restraint has never yet been used in this asylum, nor are there on the premises any special means for applying it. My opinion, drawn from personal observation, is, that its application may always be done without; that the cases in which a moderate amount of it might be beneficial are very rare, and that if used at all it should be in the mildest possible form: and only as an adjunct to the unremitted vigilance of attendants. Among the 258 cases that have been under treatment in the asylum, there is but one in which I think it might have been useful."

We hope this gentleman will not be offended at our placing him in the present class. Without any reference to the number of patients under care, if a superintendent acknowledges that he is obliged to use mechanical restraint even in one case, we consider ourselves justified in placing his name among the advocates for the partial application of restraint in special cases.

Dr. Thurnam, of the Wilts County Asylum, after stating that there is literally no instrument of coercion in the institution, observes that he "is not of opinion *that in no possible case is it justifiable or proper to have recourse to personal restraint.* There are, he believes, rare instances in which it may be needful temporarily to resort to it; in order, for example, to prevent the removal of surgical apparatus, or in some anomalous cases of perverted instinct, among which may be adduced that now and then observed, of the patient manifesting a determined propensity to gnaw his own flesh. Such instances are, however, truly exceptional; and the writer entertains a very strong conviction that the officers and attendants in an asylum should be trained to the habitual disuse of mechanical restraint, and that it should be on no account resorted to by the medical officer in charge, except upon very grave deliberation, and after the failure of all other methods."

Mr. S. Hill, of the Yorkshire, North and East Ridings' Asylum, thus describes his mode of restraint:—"A spencer, made of thick linen, to button or lace behind, with sleeves ending in pockets, which latter are sown to the lower and front part of the body of the spencer, answers very generally, and is in use in this asylum for both sexes, when all other means have failed to tranquillize dangerous, destructive, or suicidal patients."

Mr. T. Green, of the Birmingham Borough Asylum, says—"I have not used mechanical restraint at all, though I am not, with some, prepared to say that it is in all instances injurious. On the contrary, I think that there are cases in which its employment is not only justifiable, but beneficial."

Mr. Stainsbury, of the Bristol Lunatic Asylum, admits:—"The waistcoat and wrapper are sometimes used; the former for the most violent. We do not, in its use, allow the arms to be crossed on the body, as such constraint tends to congestions, but prefer allowing a certain amount of freedom to the arms by securing the sleeves to each side of the bedstead when in the recumbent posture, which has proved to be equally safe, and must be far less irksome than crossing the arms on the body."

Mr. W. F. Casson, of the Hull Asylum, says, "that mechanical restraint and seclusion are both occasionally adopted; the former chiefly by means of a strong ticking or fustian dress, the sleeves of which are attached to the sides, the hands and arms being, as it were, in deep pockets. This method is employed when a constant tendency exists to destroy the clothing, &c., or to undress, employment failing to produce the desired effect. I am not an advocate for the total disuse of mechanical restraint and seclusion, having found them useful; both, however, are sparingly employed, the latter more frequently than the former."

Dr. Formby, of the Liverpool Lunatic Asylum, says:—"We are ready to employ mechanical restraint where it is necessary to promote the welfare of the patients, yet that such cases are extremely rare, and only allowable where other means fail to attain the desired object—viz., the care, comfort, and cure of the turbulent insane."

Mr. Gibson, of Bethel Hospital, Norwich, says:—"The rule of treatment is on the principle of non-restraint. But occasionally violent patients are prevented doing harm to themselves or others by a strap fastened round their waist or wrist, and are also secluded in their own rooms when very noisy. We have no padded rooms in the establishment; but mechanical restraint is quite the exception, and not the rule, here."

Mr. Allen, of Warneford Hospital, admits that he has used mechanical restraint in the treatment of his cases under the following circumstances:—"1. Female, one night only, to prevent her being utterly naked by the destruction of her night apparel and bed-clothes. 2. Female, once only for nine hours, to prevent violent attempts to injure herself in a paroxysm of acute mania. 3. Male, once only for two hours, for repeatedly tearing his own clothes to pieces. Five patients have been placed in seclusion for short periods:—1. Male, twice, for mischievous destruction of everything within his reach. 2. Male, once for incessant shouting and blasphemous swearing. 3. Male, once, for extreme violence and assault. 4. Male once, for the same. 5. Female, occasionally, for the habitual use of indecent and disgusting language."

Dr. Edward Simpson, of the York Hospital, admits that he has adopted a mild form of mechanical restraint in rare and exceptional cases.

Mr. Kitching, of the Friends' Retreat, York, says that the asylum, under his care, "has not considered it wise to pledge itself to the non-restraint practice as a principle, conceiving that there may still be exceptional cases in which mild restraint is the best and kindest, as well as the most scientific mode of dealing with them."

Mr. James Phillips, of Bethnal House, says:—"Mechanical restraint is never employed with the idea of diminishing excitement, or as a precaution against violence or suicide; never as a mere punishment, or with the purpose of saving trouble to the attendants, by diminishing their vigilance, or to render their persevering efforts of persuasion and kindness less necessary. I am not one of those who have convinced themselves that, in the practical treatment of insanity, it can be entirely disused."

Mr. M'Clure, of Earl's Court House, uses mechanical restraint "in extreme cases of maniacal violence, where the patient is quite incoherent, and where I considered the patient in danger of sinking from the exhaustion brought on by the violence."

Mr. Roy, of Hammersmith, says:—"More than one case has come under my notice, when, from the patient constantly getting out of bed at night, sleep could only be procured when restraint was used."

Mr. Paul, of Camberwell House, has used mechanical restraint in one case:—"The patient to whom the camisole was applied was a female labouring under a violent paroxysm of maniacal excitement, in which a suicidal propensity was strongly marked, the attempts at self-destruction being of an unusually sudden and dangerous character, and the severity of the symptoms being obviously increased by the presence of attendants."

Dr. Sutherland says, he agrees with Dr. Conolly, "that restraint cannot be dispensed with in all cases, and under all circumstances, with benefit to the patient."

Mr. Bush, of Clapham, says:—"My own observations lead me to believe that there are cases in which the application of the waistcoat is unmistakably beneficial and curative in its effects, inducing sleep where other means have failed; and also in some cases, where sudden and furious paroxysms show themselves, I consider it less hazardous to the patient than prolonged struggling with attendants."

Dr. Monro is an advocate for the use of mechanical restraint, and has used it "in the cases of a few female patients more especially, and with a view to the prevention of violent outbreaks or mischievous conduct. These instances, however, have been of short duration, and their object simply the prevention of injury to themselves or others."

Messrs. Bowling and Halford, of Normand House, admit to have used mechanical restraint in the casc of a patient who "is exceedingly dangerous, filthy, and indecent. She is kept in a room by herself, with a small portion of the garden railed off for her use. The only restraint used is the occasional muffling of the hands and confinement of the arms, to prevent her picking up and swallowing stones, broken glass," &c.

Dr. Oxley, of Hackney, says:—"In some cases, however, it has been necessary to have recourse to restraint, and I have found it beneficial in aiding medical treatment."

Dr. Forbes Winslow says:—"Mechanical restraint is rarely resorted to in the establishments under my management, except when its application is rendered absolutely necessary for the protection of human life, and the prevention of habits subversive of health, and obviously inimical to recovery. For many years, the strait-waistcoat has not, excepting in one or two cases, presenting peculiar and anomalous features, been used in either of the asylums under my care." He then cites a case in illustration, and observes:—"In this, as in every other case, no servant of the establishment has the power of applying any kind of mechanical restraint without the sanction of myself or my assistant medical officer." He subsequently states that, "as a curative process of treatment, gentle and modified mechanical restraint is occasionally beneficial. I have no hesitation in recording this to be my deliberately formed opinion. Patients have often expressed a wish to be placed under mechanical restraint, should I, in my judgment, believe that they would, when much excited, commit overt acts of violence, and be dangerous to themselves and others. In cases like these, mechanical restraint may, for a short period, be applied, not only without detriment, but with positive advantage as a curative process. Several instances illustrative of this fact have come under my observation. I have seen cases in which no food or medicine could be administered without subjecting the patient to restraint. In these cases, if all idea of cure had been abandoned, and I could have reconciled it to my conscience to allow the disease to take its uninterrupted course, and have permitted the patient to exist upon the minimum amount of nutriment, and take no medicine, all restraint might easily have been dispensed with; but considering the cure of my patient paramount to every other consideration, I had no hesitation as to the humane and right mode of procedure. Whilst recording these particulars, conclusively demonstrative, according to my humble judgment, of the propriety and necessity of mechanical restraint, under peculiar and pressing circumstances, I wish the Commissioners in Lunacy distinctly to understand that I have no hesitation in admitting that, as a general principle in treating the insane, mechanical restraint and prolonged seclusion should undoubtedly be dispensed with. In the management of the insane, and in the conduct of asylums, both public and private, the principle of treatment should consist in a full and liberal recognition of the importance of extending to the insane the maximum amount of liberty and indulgence compatible with their safety, security, and recovery; at the same time, subjecting them to the minimum degree of mechanical and moral restraint, isolation, seclusion, and surveillance, consistent with their actual morbid state of mind at the time. It is also necessary to bear in mind as an essential principle of curative treatment, the importance of bringing the insane confined in asylums, as much as possible, within the sphere of social, kindly, and domestic influences. In many cases, isolation, seclusion, and an absolute immunity from all kinds of stimuli, physical and mental, are, during the acute and recent stages of insanity, indispensably necessary to recovery; but in certain forms of melancholia, monomania, and in some chronic morbid states of mind, no mode of moral treatment is productive of such great curative results as that now referred to. I need not observe, that this system of treatment cannot be adopted except in those establishments where there is an active, experienced, and intelligent resident medical officer, who fully appreciates the great value of such homely family influences upon the minds of the insane. In our moral treatment, do we not occasionally exhibit an excess of caution, and exercise, with the best and kindest intentions, an undue amount of moral restraint and vigilance? I think we may sometimes err in being a little too distrustful of the insane. Whilst urging the necessity, in certain forms of morbid mind, of great and constant watchfulness, particularly in cases of suicidal monomania, and recent and acute attacks, I would suggest, to those having the management of asylums, the necessity, with the view to the adoption of a curative process of treatment, of placing more confidence in those entrusted to their care, and of allowing the patients a greater amount of freedom, indulgence, and liberty than they at present enjoy in many of our public and private asylums. In many phases of insanity in which confinement is indispensable, the patient's word may fully be relied upon; and under certain well-defined restrictions, he should be permitted to feel that confidence is reposed in him, and that he is trusted, and not altogether (although in confinement) deprived of his free and independent agency. I feel quite assured that a judicious liberality of this kind will be generally followed by the happiest curative results, and greatly conduce to the comfort and happiness of the patient. Patients should be permitted occasionally to attend divine worship out of the asylum, when circumstances do not contra-indicate this practice; they should be allowed also to walk out of the confines of the asylum, to attend places of amusement, visit scientific exhibitions; and the resident medical officer should make himself their friend and companion; thus inspiring them with confidence in his skill and kindly intentions, and reconciling them to the degree of moral restraint to which they may be unavoidably subjected."

Mr. Nicoll, of Hanwell, without denying that mechanical restraint is necessary, says it "is very rarely called for, and that seclusion is seldom necessary."

Mr. J. W. Holgate, of Hendon House, Middlesex, says:—"In two cases of maniacal paroxysm, it was found necessary to control the patients very temporarily, by means of a long-sleeved vest; a course, I conceive, more humane and preferable to a resort to the padded room (particularly in cunning and suicidal patients); patients can be more readily fed, an all-important matter; medical means, &c., more readily applied, as cold lotions to the head, &c.; and the patient's habits do not necessarily degenerate to those of the mere animal; and I may further add, that by occasional judicious mechanical restraint, the patients are less irritated and less likely to be injured; the attendants, also, are less likely to be worn out and exhausted."

Dr. Armstrong, of Peckham, says:—"Restraint is but seldom used now in this asylum: nevertheless I am of opinion that there are some cases where the application of restraint is essential for the well-being of the patient; but such cases are of rare occurrence."

Dr. Atkins, of Stoke Newington, says that—"Some instances have occurred in which I have deemed it advisable and even necessary to have recourse to mechanical restraint. Cases have occurred in which patients would have been in a state of nudity from various causes (more particularly from the occasional practice of destroying their clothing), but for the use of some prohibitory measure, such as the jacket or gloves."

Mr. W. T. Spencer, of Stoke Newington, says that "mechanical restraint has been, and is being, considerably diminished, but notwithstanding every effort and desire to effect its total abolition, it is in some cases found to be impracticable."

Mr. Glenton, of Bensham Asylum, speaking of mechanical restraint, says:—"Exceptional cases occur in which it is very difficult and even dangerous to dispense with it. I may cite the case of J. S.; he is subject to very violent paroxysms of epilepsy; immediately after the fit he becomes homicidal, and being a strong and extremely muscular young man, no single attendant can cope with him, or restrain him from doing that which might have a fatal issue. My predecessor nearly lost his life during one of these paroxysms. He fastened on his throat with both hands, and but for timely aid he would have perished. I have myself seen him attack an attendant in the same manner. In such a case I think coercion is unavoidable; it would require the constant attendance of two men to prevent fatal consequences, and also to have two separate entrances to the room where he is placed; there is besides the risk of injuries during a struggle."

Mr. Dairs, of Wreckenton, observes—"I am inclined to believe that mechanical restraint can scarcely be entirely and unreservedly abolished, for cases frequently occur, where a certain degree of restraint to prevent the patient injuring himself, or those around, is unavoidable; and I am induced to consider restraint by mechanical means more effectual and less irritating to the feelings, as well as more easily and readily obtainable, than any other."

Dr. Barkus, of Gateshead, says that—"In some cases, when the patient is very violent to himself and others, he is placed in the strong-room for a short period; if he tears his clothes, or strips himself naked when other measures are of no avail, handcuffs are used. These methods are generally effectual for the purposes aimed at."

Dr. Griesbach, of Dunston, has "found it necessary to apply restraint in two or three instances, to prevent the patients inflicting injuries on themselves. In each case, the method of restraint has been by a light strait-waistcoat, loosely applied, so as not to interfere with respiration, or cause the slightest pain; and in those patients of destructive habits, we have substituted the ordinary blankets, stoutly bound at the edges with jean or bedticking, and also their personal clothing is now made with stouter material than those patients who are not so destructive. These means we have invariably found to supersede the necessity for restraint."

Mr. Tomkin, of Witham, uses mechanical restraint in cases of "acute mania, strong suicidal tendency, showing great violence to others, breaking windows, a constant habit of tearing clothes, and burning them, &c."

Mr. Cornwall, of Fairford, says that he would not again resort to mechanical restraint, "unless under the most pressing emergency."

Dr. C. M. Burnett, of Alton, says:—"On the subject of mechanical restraint, I am still of the same opinion I expressed in 1848; viz., that as a remedial means it has its use, like all other means, which cannot either with safety or advantage be put aside; and it would be a parallel piece of wisdom to denounce the hygienic or the therapeutic treatment of the insane, simply because it is possible to fall into grave abuses in the employment of such means. I do not think this fact has been put candidly and dispassionately forward by writers, who say that it is possible to treat the insane entirely without mechanical restraint. They deceive themselves by supposing that the principle of mechanical restraint is done away with, simply because it has been transferred from the person of the lunatic to the building or room in which he is confined. The question at issue does not involve the principle, and is simply one of degree. And upon this point, I am quite decided that the necessity for resorting to mechanical personal restraint is by no means so great as was formerly supposed to be necessary, or really was necessary; and several circumstances conspire to effect this; but three may be particularly named:—1st. The more frequent residence of a medical superintendent, and, consequently, the more complete supervision in asylums; 2d. The improved condition of the attendants; and 3d. The improvement in the general treatment of the insane."

Mr. Millard uses mechanical restraint in extreme cases as follows:—"1. That of furious mania, where the patient strikes and bruises his own body. 2. In a case of pernicious practices, which are humiliating and degrading, and a hidden source of insanity, more frequent than is supposed. 3. In a case of one who feeds on disgusting matter, which destroys life. 4. In a case where a patient refuses to take food and his life is endangered by starvation, and you cannot feed him (on account of his resistance) without mechanical restraint."

Mr. Smith, of Hadham:—"In cases of violence mechanical restraint would be resorted to, in the strong and conscientious conviction that it is most humane, and by far the most beneficial."

Mr. Young says he adopts non-restraint so far as he believes it possible to do so in a small private asylum.

Mr. Ainsworth considers it necessary to use restraint, he says:—"Some of the patients are so violent and unmanageable as to render this, in some instances, at times absolutely necessary, both on account of their own safety and comfort, as well as for the safety of those about them."

Dr. Noble, of Manchester, is "decidedly of opinion that, in certain cases, some humane contrivance for mechanical restraint is preferable to a struggle between patient and attendant. The latter proceeding, I am led to think, irritates and excites by the sense of personal antagonism which it creates. In the very few cases in which I have seen employed the mild system of physical coercion described, I have not observed the production of any injurious sense of humiliation or degradation."

Mr. Whitehead, of Warrington, observes—"Mechanical restraint and seclusion are seldom employed, and where it has been the case that either one or the other has been adopted, it has been with the view of preventing the patient from injuring himself, where other means have been tried and failed, or as a means of keeping him in bed, and so give him the benefit of a few hours' rest; for I consider that restraint, judiciously employed, acts as an anodyne, and proves a source of great comfort to the patient. It is not the use of anything which makes it objectionable, but its abuse. The gloves are the only restraint used here."

Dr. F. Willis, of Shillingthorpe House, says:—"From my own experience, and that of my predecessors, who were most successful in their treatment, I consider mechanical restraint in the feverish stage of the disorder, when a patient, through his fever and restlessness, cannot govern himself, a most merciful and beneficial means of cure, combined of course with medicines calculated to remove these symptoms."

Mr. Landor, of Heigham, Norwich, says :—" Restraint and seclusion are both nearly abolished ; of the first, there has been only one instance in this house for four years, and there is a general opinion that seclusion and padded rooms are needless. Air and exercise are constantly required, and are the most useful parts of treatment: occupation is most desirable, but is the most difficult to obtain in private asylums, because the class of people in them is one unused to manual labour, and to whom any active bodily useful occupation is repugnant."

At Heigham Hall, Norwich, under the joint care of Mr. Nicholls, Dr. Ranking, and Mr. Watson, mechanical restraint is resorted to in the treatment of certain forms of insanity. These gentlemen observe—" With reference to the mooted question of the total abolition of personal mechanical restraint, we beg to state that we acknowledge to the fullest extent the advantages, as well as the moral obligation, to dispense with the frequent recourse to restraint of any kind. At the same time, we regard the entire and unconditional abolition of simple mechanical restraint as a piece of psychological quackery, well adapted to catch the unreflecting sentimentality of the vulgar, but rarely, we have reason to believe, carried out to its fullest extent even by its warmest advocates. The occasional use of the muff we regard as indispensable in certain cases, and we think it at all times merciful in comparison with the horrors, physical and psychological, of a padded room, where the patient is left to himself for hours, and alone. At the same time that we sanction the occasional use of such means of restraint as the muff and waist-belt, we most distinctly pronounce that it is not with the object of economising attendants, but from a conviction that it is for the advantage of the patient. We have, in fact, yet to learn that such restraint of a violent patient is more irritating to him than the continual jostling and struggling with two or three attendants. There can be no comparison in its moral effect; the first method gives cause for violence and irritation to both patient and attendants; the other satisfies the patient that, however much he may object to it, there is an authority superior to his own, and that he must learn obedience and self-control. We must again repeat that it is upon principle, and not for mere convenience, that we continue to employ a certain amount of carefully applied mechanical restraint."

Dr. Mackintosh, of Newcastle-upon-Tyne Asylum, admits that "instances do occur, wherein mechanical restraint becomes a necessary and salutary agent of cure. I am of opinion, after a practical and almost daily observation of above 20 years' residence in an asylum, that the non-restraint system has many decided advantages; but it has a limit beyond which it is dangerous to go; and cases do occur wherein mechanical restraint really proves salutary, and is the only means of relieving the system and preserving life. I look on the non-restraint system as generally sound and practicable, but the total disuse of it I consider as unsound, and sometimes fatal in practice. The abuse of restraint all rational men must condemn, but on that account to fly to an opposite and most dangerous extreme, from fear of doing one's duty, or to establish a principle, is what no honourable mind would sanction or encourage."

Mr. Mallam, of Hooknorton, in speaking of restraint, says that "there are cases in which it is necessary for the benefit of the patient, and the safety of those who have the charge of him."

Mr. Norton, of Tenby, says:—"As to restraint, it is both unnecessary and hurtful in all cases except in acute mania, where I feel convinced it is in some cases, and at certain times, absolutely indispensable; thus in cases where patients will sit up all night tearing their bed-clothes, bedaubing themselves and apartment with excrement and the like, they are better invested with a garment which prohibits the use of the hands; they will the sooner lie down, and sleep overcome them, than if allowed to irritate themselves by tearing and knocking about all night. In short, with careful and kind conduct on the part of attendants, not one case in 100 requires restraint in the day-time."

Drs. Francis and Charles Fox, of Brislington House, Bristol, speak as follows on the subject of restraint:—"The average number of patients in the Asylum for each week has been 86. The average number under seclusion for each week has been 2. The average number under restraint for each week has been 2. We must observe that the cases of seclusion and restraint were generally only for short periods during the day. We are of opinion that any system professing to reject all mechanical restraint in the treatment of insanity, would be injurious to the insane; but we think it right that satisfactory reasons are required to be entered in the weekly reports whenever occasion for such restraint arises. With respect to seclusion, instances so often occur when the removal of a patient to a separate room will tranquillise excitement in the case itself, whilst it contributes to the comfort of the rest of the community, and the measure itself is often so acceptable to the patient, that we consider it should not in such cases form an entry in the weekly report, but merely be recorded in the patients' case-book. We believe that the same moral, physical, and other causes which have gradually induced a more asthénic type in bodily diseases, may have effected a similar change in cerebral disorders, and that from hence in part results the less frequent occurrence of high maniacal excitement; but we also ascribe much of this mitigation to the more enlightened principles of treatment which have been adopted. We consider that the regulations by which a community of insane persons is conducted, should be assimilated in a great measure to those of other associated bodies. The recognition by the insane of an authority to which they must defer, with the knowledge that they have a power of appeal against an undue exercise of it, is of much value, the maintenance of civilized habits, out and in-door pursuits adapted to the tastes of each individual; social intercourse with the medical superintendents, their families, and the chaplain, and especially a regular participation in the observances of religion, afforded by the daily public ministrations of a chaplain, form the basis of the system of moral management adopted by us. We attach a high value, even in incurable cases, to the consoling and restraining influence of religion."

Mr. Terry, of Bailbrook House, Bath Easton, says:—"We do not profess to have entirely abandoned the use of mechanical restraint; we have always considered it as a remedial measure, to be applied solely by medical order; but we employ it only in those cases in which some manual coercion would otherwise be necessary, such as where a sudden and violent desire for self-destruction occurs in paroxysms. In such cases, I believe that some light form of mechanical confinement is less irritating to the patient, and much more safe than forcible restraint by the hands of attendants, however imposed."

Mr. Gillett, of Taunton, although he rarely uses restraint, observes that—"Cases, however, occur at times in which, in my opinion, it is advisable to have recourse to it, and these are where the patients will expose the person, and, unless prevented, will not keep on any clothes; others, where the patient is wilfully destructive, and is determined to do mischief either to himself or to those about him. Restraint, however, should only be resorted to when other means fail, and this in cases of extreme necessity."

Mr. Woody, of Tamworth, employs mechanical restraint "only in cases of acute mania, and only in those cases in which danger of injury is apprehended to the patient himself. Such restraint has always been limited to the use of a strait-waistcoat, but never resorted to when the watchfulness and care of one or more attendants has been thought sufficient to protect the patients themselves from injury."

Messrs. Furnival and Charles Summers, of Great Foster House, Egham, say that for many years they have not employed mechanical restraint, "unless in strongly suicidal and violent cases, and where the patients would otherwise injure themselves. We have at this time but one patient who is subjected to restraint, and that is always of the simplest and mildest kind; viz., the hands confined with the muffs or gloves, at night only, to prevent her from tearing her dress or bed-clothes, or doing injury to herself."

Mr. Stedman, of Guildford, is occasionally obliged to use mechanical restraint, and Mr. George Stillwell, of Epsom, says that he has not used restraint, "except in two severe cases of acute mania, when a linen waistcoat was employed, as medical means, to prevent and restrain the violence of the paroxysms."

Messrs. Newingtons, of Ticehurst, Sussex, say that:—"Occasionally a patient is admitted in a maniacal state, when the loose camisole, with long sleeves, is placed on him, until the violence has subsided; and we find it a more harmless mode of securing him from injury than the mechanical restraint of men's hands. Such cases are necessarily rare in a private asylum; this violent stage of mania belonging rather to the acute form of encephalitis, and being usually treated in private until the more permanent symptoms of insanity have indicated themselves. There is one class of patients with whom, contrary to our general rule, and more especially of late years, we have adopted a system of mechanical restraint; and we believe that, in these cases, beneficial results have been derived. They are cases of pernicious practices, which no amount of watchfulness can prevent. The wrists are fastened by a soft strap to the sides of the bedstead. As a general rule, we have found, that where mechanical restraint is absolutely necessary, the habit which called for it has shortly ceased when the patient has found that a restraint is placed on his actions. The result of our observations and experience at Ticehurst is, that it is impossible to establish a fixed rule, forbidding all mechanical restraint; but it will be seen by the foregoing remarks, that very much is to be effected without resorting to such measures, provided the number of attendants is ample. A patient, cheerful, and respectful behaviour on the part of an attendant, indulgence towards harmless caprices, but steadiness in not permitting what would prove injurious; change of attendant, where an obvious antipathy has arisen, and various forms of moral control, will often accomplish what no amount of mechanical restraint will effect, by calming a violent patient, and thus promoting his chance of recovery, while at the same time he is equally secure from mischief."

Mr. Bodington, of Driffold Asylum, remarks:—"That the theory of total non-restraint, so loudly proclaimed and upheld by some practitioners, has resulted, on the whole, in much advantage in the treatment and cure of lunacy, there can be no doubt; but, like all imperfect theories, when carried into practice, the good effected has not been unaccompanied with serious concomitant evils, plainly pointing to a modification of the system as the best for general adoption. Properly modified and regulated, the abuses which formerly existed under the restraint system may be for the most part swept away; and at the same time the dangers and difficulties arising from the total absence of all restraint at all times unexceptionably, may be avoided. I cannot but consider the doctrine of total non-restraint to be an ultraism which overshoots the mark, and goes beyond the truth. It becomes, then, a question as to the best method of meeting, resisting, and overcoming the propensity to attack and destroy, which commonly appertains to mental derangement, and which, under certain forms of the malady, in some way or other displays itself. I have no hesitation in declaring my entire conviction that the use (taking care that there should be no abuse) of instruments of restraint, properly adapted, is the most efficacious and merciful way of meeting the difficulty. I hold the clamour, the agitation, or the remonstrances against instrumental restraint to be good and tenable only as against the abuses of it, and that when carried so far as to overturn the use of that system, they tend to substitute other abuses for those they would remove. There are the cases of the lunatics who will not keep their beds, but will be up even all through the night, and in severe frosty weather are in danger of being frost-bitten. No personal efforts of an attendant can be effectual in remedying this evil. The system of total non-restraint leaves these cases quite unprovided for. It is impossible to meet them otherwise than by a mild and judicious application of instrumental restraint."

Mr. Warwick, of Laverstock House, near Salisbury, says:—"As I consider the milder forms of mechanical restraint to be of service in some cases of insanity, I still occasionally employ them. I advocate plenty of exercise in the open air, out-door games, walks in the grounds, rambles about the surrounding country. I promote the breeding and keeping of domestic animals by patients; and also the cultivation of flowers; I encourage music, singing, drawing, painting, fancy works, and the usual recreations. Billiards, chess, backgammon, cards, reading, &c."

Dr. Nash, of Kingsdown House, Box, says, that "mechanical restraint is very rarely used in my establishment. I have found it necessary on a few occasions, in extremely violent and dangerous cases, to use a waistcoat until the violence of the paroxysm had subsided, but have never continued it longer than absolutely necessary for the safety of the patient or others, as the case might be. I have always tried gentle means in the management of the insane, and feel sure much more may be done with kindness than in any other way, except in very violent and dangerous cases."

Mr. Anningson, of Kingston-upon-Hull, relates the case of one lady who, "with but few intervals, was obliged to be kept constantly under restraint, in consequence of her determined violence and destructive habits, until the 31st of August, 1853, when she was removed to another establishment. Since that time, no patient has been subjected to either restraint or seclusion."

Dr. P. Smith, near Leeds, says that:—"Exceptional cases of mania exist where the use of mechanical restraint for a short period is followed by the most beneficial effects, where the patient, possessing a certain control over his actions, is yet deficient in the wish to exert it. In these cases, a short period of 'duresse' excites the dormant power of self-control, as a prevention of further restraint."

Mr. Allis, of Fern Hall, near York, remarks, after referring to some cases in which he has used restraint, that he has "no hesitation in saying that the employment of such restraint was beneficial to the patient. One of the cases is now in the house, and the use of such restraint has never for a moment interfered with the kind feelings which have always subsisted between the patient and the managers of the institution."

Mr. Metcalfe, near York, states that he would not have recourse to mechanical restraint "for any case of mischief, only where danger to the patient or others exists, and this of extreme nature; were it used, the medical treatment would not be more carefully weighed; and I should, as hitherto, only use it myself, keeping the means as the drugs are kept, and classing them as aids with any nauseous medicine, or powerful remedy, not to be trusted in unskilled hands."

Mr. Nelson, near York, declares that he is "an advocate of mechanical restraint, and considers it, when properly used, a powerful remedial agent; it is a promotive of comfort to the patient, a preventive of suicide and injury to others, and an adjuvant to medicinal treatment in procuring quiet, and consequently sleep, and I am borne out by the testimony of some of the patients after recovery. One (a case of acute mania) stated that he could not sleep, except when restrained; another (a case of intermittent mania) was so conscious of the comfort, that he wished to be restrained at the approach of the fits."

Mr. Atkinson, of Heworth Asylum, near York, says that he has entirely abandoned restraint, "except in one single instance, the case of a female, who was very destructive during the night. A single light glove put on the right hand had the effect of preventing her from destroying her clothes. As to seclusion, I have never employed or tried it, in any case at Heworth. When very violent, I have had recourse to a few doses of Ant. Tart., which I have invariably found serviceable."

A number of gentlemen assume the title of advocates for non-mechanical restraint, but who consider it necessary to forcibly confine the limbs of patients in certain surgical cases.

Dr. Robert Boyd, of the Somerset Asylum, says:—"In reply to your question of mechanical restraint, I beg to state that nothing has ever been provided or used for that purpose in this institution. In the last five years there have been six or seven cases, chiefly under surgical treatment, in which it was essential to prevent the patients removing the dressings; and the wrist was accordingly, so far as necessary, confined by a handkerchief."

Dr. Harrington Tuke, of Manor House, Chiswick, admits that he has been obliged to use mechanical restraint. In this case he says:—"I employed restraint for a few nights with most satisfactory results; but I had recourse to it with great reluctance, and only after frequent consultations with the friends and former medical attendant of the patient. I do not often use the padded room, and never in melancholia or suicidal mania."

Dr. Conolly has recorded his experience at some length, and although of course it is well-known what his views are on the question of mechanical restraint, he nevertheless admits that he has found it necessary to use it in a few surgical cases. When speaking of the former condition of the asylums in this country, he says:—"The disuse of mechanical restraints of all kinds has been productive of an incalculable amount of advantage to the insane. The general tranquillity, comfort, and satisfaction visible in all well-conducted asylums, public and private, attest this in the strongest manner. Fewer accidents occur; revenge is seldom excited in the minds of the patients; scenes of violence are seldom or never witnessed; the patients manifest no terror, and, on recovery, retain no sense of degradation; often after leaving the asylum, coming to it again as voluntary visitors to associates and friends, of whose good offices they are fully sensible."

But we would ask Dr. Conolly, whether the great improvement that has taken place in the management of public and private asylums is not in *some measure* to be attributed to the more general prevalence of enlightened views with reference to the pathology and curability of insanity? We do not think it fair to ascribe the general amelioration that has taken place in the condition of the insane *solely* to the inculcation of opinions adverse to the use of mechanical restraint.

Mr. Parsons, of Bristol, says that "mechanical restraint is never employed, except when it may become necessary in the course of surgical treatment, to prevent, for instance, the forcible removal of dressings from a wound."

We proceed with our analysis of those who represent that mechanical restraint is not resorted to in the asylums under their management, but who express *no opinion* upon the abstract question. We can well understand that a medical superintendent of an asylum may be in a position to say, that no mechanical restraint is at the period of the making his return to the Commissioners used, but who nevertheless is of opinion, that cases do arise, for the safety and treatment of which it would be necessary. Dr. Begley, of Hanwell, says as follows:—"With reference to the disuse or employment of mechanical restraint and seclusion, I have to state, that the former has not been used in this asylum for several years, and that on the male side the latter is resorted to chiefly in cases of excitement premonitory of, or consequent upon, paroxysms of epilepsy. Seclusion is also occasionally employed in cases of recent, and in those of recurrent mania, usually, however, only for brief periods; exercise in the open air, with an attendant, sometimes with two (according to circumstances), being generally found efficacious in soothing such patients, and with the aid of medicinal and dietetic remedies, procuring refreshing sleep for them. In the irritability, too, manifested by persons affected with general paralysis in the last stage, and attended with much debility, it is customary, when the weather does not admit of their being drawn about the grounds in Bath chairs, to seclude these in padded rooms, for protection against injuries by falls, &c."

Mr. Stocker, of Guy's Hospital, says:—"All restraint has been removed (except restriction to the room of the patient on the occurrence of violent paroxysms of mania); and this liberty has been followed by most marked improvement in the general condition and conduct of the patients, many of whom, having previously conducted themselves with great violence, and contracted very offensive habits, have, since the adoption of the non-restraint system, been much more quiet, cleanly, and orderly."

Dr. Steward, of Southall, adopts the non-restraint system.

Dr. Wood, of Kensington, says that he has used no mechanical restraint since his connexion with this establishment, but he makes the following important admission, that "it is impossible to deny that, notwithstanding an increased number of attendants, casualties will occur more frequently where restraint is altogether disused."

Mr. Rumball, of St. Alban's, may be placed in this class.

Mr. Slatebrook, of Brook Villa, Liverpool, says,—"Since my appointment, there has not been any mechanical restraint. In some instances we have tried half an hour or an hour's seclusion; in other instances, seclusion in the padded room. Seclusion has produced a good effect; it has calmed violence, changed the obstinate, silenced the noisy, soothed the irritable, and induced sleep in the restless; in some, the result has been such, that a mere reference to it has had the desired effect."

Mr. William Cooper, of the Norwich Infirmary, says:—"Every species of mechanical restraint is abolished, and recourse but rarely had to seclusion. When it becomes necessary to seclude, it is seldom needful to carry it beyond a few hours, and it is principally adopted where a patient requires to be removed as a means of protection to the others. The most refractory are sometimes removed to their dormitories for a short period, and this temporary banishment is found useful. The padded rooms with which the institution is provided are but seldom used, and only when the patient is uncontrollable by any moral agency. The class of patients most frequently subjected to this species of control are those who, when admitted, are in a state of furious mania; and much benefit is frequently derived by a short detention in this room: it removes them from all sources of external irritation, and except where fear is induced by it, which I have occasionally seen (especially with females), it operates in producing a calm and quiet most desirable of attainment. In the early stages of acute mania, rest and seclusion, removal from all excitement and from every predisposing cause, constitute the principal method of cure adopted."

Mr. Pritchard, of Northampton, does not find it necessary to use mechanical restraint in the treatment of his patients.

Mr. C. Williams, near York, says,—"There has been no mechanical restraint or seclusion employed since I have been in attendance,—viz., two months."

Mr. Langworthy, of Plympton House, gives a qualified opinion on the subject, and says that, although he never uses mechanical restraint, "in some suicidal cases it may be desirable to do so, unless implicit confidence can be placed in the servants."

We have now fairly laid before our readers an impartial summary of the opinions of some of the principal gentlemen engaged in the practice of lunacy in this country, on the subject of mechanical restraint. It is but right that we should state that many of the superintendents connected with several of our private and public asylums, have made no replies to the circular of the Commissioners; however, the evidence is considerable upon the point, and having given a digest of it, we leave our readers to form their own conclusions upon the important point to which it refers. The Commissioners in Lunacy still entertain the opinion that all mechanical restraint in the treatment of the insane may eventually be done away with. It would appear that an influential body of men, practically engaged in the management of asylums think differently.

---

### Art. VII.—RECENT TRIALS IN LUNACY.*

